# Regional Citrate Anticoagulation and Systemic Anticoagulation during Pediatric Continuous Renal Replacement Therapy: A Systematic Literature Review

**DOI:** 10.3390/jcm11113121

**Published:** 2022-05-31

**Authors:** Emanuele Buccione, Stefano Bambi, Laura Rasero, Lorenzo Tofani, Tessa Piazzini, Carlo Della Pelle, Khadija El Aoufy, Zaccaria Ricci, Stefano Romagnoli, Gianluca Villa

**Affiliations:** 1 Neonatal Intensive Care Unit, 65124 Pescara, Italy; 2 Health Sciences Department, University of Florence, 50139 Florence, Italy; stefano.bambi@unifi.it (S.B.); l.rasero@unifi.it (L.R.); 3 Section of Anesthesiology, Intensive Care and Pain Medicine, Department of Health Sciences, University of Florence, 50139 Florence, Italy; lorenzo120787@gmail.com (L.T.); tessa.piazzini@unifi.it (T.P.); zaccaria.ricci@meyer.it (Z.R.); stefano.romagnoli@unifi.it (S.R.); gianluca.villa@unifi.it (G.V.); 4 ASL02 Lanciano-Vasto-Chieti, 66100 Chieti, Italy; carlo.dellapelle@unich.it; 5 Department of Experimental and Clinical Medicine, Azienda Ospedaliero Universitaria Careggi, 50134 Florence, Italy; khadija.elaoufy@unifi.it; 6 Pediatric Intensive Care Unit, Meyer Children’s University Hospital, 50139 Florence, Italy; 7 Department of Anesthesia and Intensive Care, Azienda Ospedaliero Universitaria Careggi, 50139 Florence, Italy

**Keywords:** continuous renal replacement therapy, pediatric intensive care unit, anticoagulation methods, systemic anticoagulation, regional anticoagulation

## Abstract

Background: Clotting is a major drawback of continuous renal replacement therapy (CRRT) performed on critically ill pediatric patients. Although anticoagulation is recommended to prevent clotting, limited results are available on the effect of each pharmacological strategy in reducing filter clotting in pediatric CRRT. This study defines which anticoagulation strategy, between regional citrate anticoagulation (RCA) and systemic anticoagulation with heparin, is safer and more efficient in reducing clotting, patient mortality, and treatment complications during pediatric CRRT. Methods: A systematic literature review was run considering papers published in English until December 2021 and describing patients’ and treatments’ complications in CRRT performed with heparin and RCA on patients aged less than 18 years. Results: Eleven studies were considered, cumulatively comprising 1.706 CRRT sessions (62% with systemic anticoagulation and 38% with RCA). Studies have consistently identified RCA’s superiority over systemic anticoagulation with heparin in prolonging circuit life. The pooled estimate (95% CI) of filter clotting risk showed that RCA is a protective factor for clotting risk (RR = 0.204). Conclusions: RCA has a potential role in prolonging circuit life and seems superior to systemic anticoagulation with heparin in decreasing the risk of circuit clotting during CRRT performed in critically ill pediatric patients.

## 1. Introduction

Acute kidney injury (AKI) is a severe condition in critically ill children, affecting short and long-term patients’ outcomes [1]. Diagnosis and staging are identified by the Kidney Disease Improving Global Outcomes (KDIGO) criteria [2]. Like adult patients, the current management of AKI is based on early diagnosis, primary and secondary prevention, etiology identification with the aim of establishing pathophysiologically driven treatments, and supporting organ function [3]. In the case of severe AKI, children may need renal replacement therapy. Critically ill children with AKI can benefit from continuous renal replacement therapy (CRRT), which supports kidney function, and potentially improves short-term outcomes [4]. The *Prospective Pediatric CRRT Registry* showed that CRRT is applied in critically ill children in very different clinical settings and with extreme variability in prescription and management among different centers [5].

Indeed, the definition of best practices for CRRT specifically performed in pediatric patients is needed. Similar to other extracorporeal blood purification therapies, preventing filter clotting is significantly challenging during CRRT. It decreases treatment efficiency, increases treatment downtime, and leads to unexpected filter substitution with increased patient blood loss and health care costs [6]. Thus, efficient anticoagulation protocols are required to prevent clotting and prolong circuit life (CL). Unfractionated systemic heparin administration and regional anticoagulation with sodium citrate are applied with this aim. In adult patients, regional citrate anticoagulation (RCA) has been demonstrated to be more effective than systemic anticoagulation with heparin in reducing clotting, prolonging filter efficiency, and reducing the risk of bleeding. Indeed, the 2012 KDIGO guidelines recommend using RCA for adult patients at increased risk of bleeding and in the absence of contraindications for citrate infusion [7]. Nonetheless, few studies have investigated factors affecting CL in critically ill pediatric patients treated with CRRT [6], and limited results are available nowadays on the effect of each pharmacological strategy in reducing filter clotting in this population.

This systematic review aims to define which anticoagulation strategy, between RCA and systemic anticoagulation with heparin, is safer and more efficient in reducing clotting, patient mortality, and treatment complications during pediatric CRRT.

## 2. Materials and Methods

We performed a systematic literature review (SLR) on critically ill pediatric patients treated with CRRT. Outcomes referring to both the patient (e.g., mortality rate) and the treatment (e.g., circuit life or clotting rate), as well as treatment-related complications (e.g., electrolyte and acid-base disturbances), were compared between groups of patients undergoing systemic anticoagulation with heparin or RCA. Table 1 shows the PICO criteria used for this SLR.

This SLR was developed following the *Preferred Reporting Items for Systematic reviews and Meta-Analyses* (PRISMA) methodology [8]. Databases such as PubMed, EMBASE, CINAHL, and Cochrane Library were considered for the literature review and data extraction. Strings were developed and used for each database, using different keywords: “citrate”, “heparin”, “dialysis”, “hemodialysis”, “hemofiltration”, “kidney replacement therapy”, “continuous renal replacement therapy”, “CRRT”, “pediatric”, and “children”. No time restrictions and no filters were used, and the last search was performed on 28 December, 2021. Strings are reported as supplemental material.

Two independent reviewers (E.B. and G.V.) assessed the identified papers. Titles and abstracts were evaluated for eligibility criteria; conflicts and disagreements were discussed with all other authors, and a unified list of eligible papers was defined. Only English-written manuscripts were considered. Studies on patients affected by liver failure were excluded and those exploring intermittent or prolonged renal replacement therapy. Commentaries, letters, editorials, case reports, case series, reviews, and meta-analyses were not included in the analysis.

The quality of eligible papers was analyzed, and studies noncompliant to standard methodologies were excluded from the final analysis. In particular, observational studies (Table S5) were evaluated using the *STrengthening the Reporting of OBservational studies in Epidemiology* (STROBE) statements [9], while randomized clinical trials (Table S6) were evaluated using the *Consolidated Standards of Reporting Trials* (CONSORT) statements [10]. For those papers included in the analysis, experimental design, sample size, numbers of CRRT circuits, patients’ features, severity, circuit life span, and total CRRT duration were reported.

In order to evaluate the difference in clotting rate, the proportion of clotting and its 95% confidence interval was used for each article and pooled weighting for sample size. The I^2^ statistic was used to quantify the heterogeneity. I^2^ was calculated using the formula proposed by Higgins and Thompson [11]. Statistical analysis was performed using SAS^®^ software version 9.2.

## 3. Results

**Figure 1:**
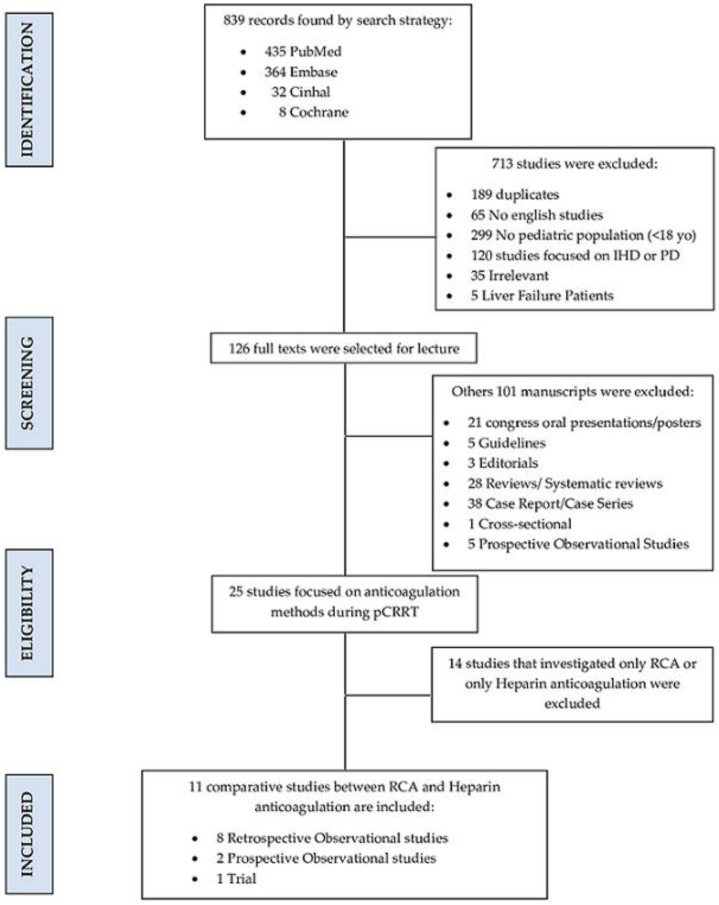
PRISMA flow chart. yo: years old; IHD: intermittent hemodialysis; PD: peritoneal dialysis; pCRRT: pediatric continuous renal replacement therapy; RCA: regional citrate anticoagulation.

Eight hundred and thirty-nine records were extracted from the literature. Figure 1 shows the selection process. Eleven studies comparing RCA with systemic anticoagulation using heparin during pediatric CRRT were ultimately considered (Table 2).

The studies included in this SLR comprise 1.706 CRRT sessions; 62% (*n* = 1.058) of these were managed using systemic anticoagulation with heparin, while 38% (*n* = 648) used RCA.

### 3.1. Circuit Life and Clotting Rate

Ten of the eleven selected studies report CL or clotting rate as treatment outcomes [13,14,15,16,17,18,19,20,21,22] (Table 3). Eight studies reporting CL as treatment outcomes, and cumulatively counting for 1550 procedures, consistently identify the superiority of RCA over systemic anticoagulation with heparin in prolonging CL. Mean and standard deviation or median and interquartile range of the resulting CL are reported in Table 3.

**Figure 2:**
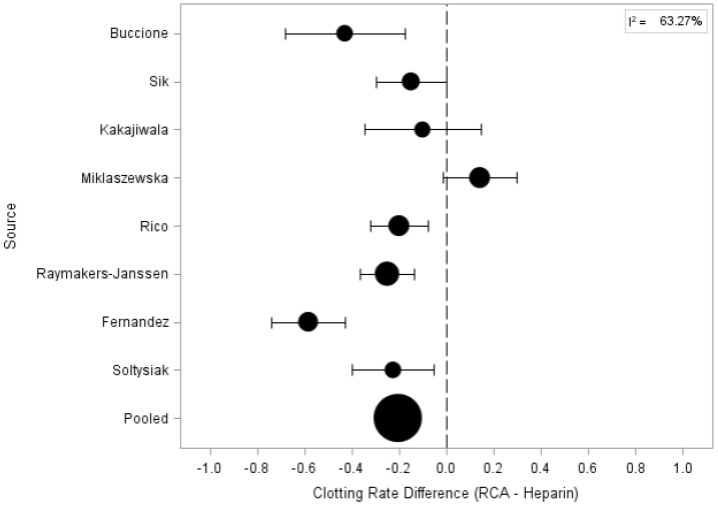
Forest plot for clotting rate difference between regional citrate anticoagulation vs. heparin anticoagulation.

Among the eight studies reporting clotting rate as treatment outcome, only one study (performed on 150 procedures) reports a higher clotting rate for RCA sessions [17]. The remaining seven studies (cumulatively considering 847 procedures) identify systemic anticoagulation with heparin as associated with a higher clotting rate [13,14,15,16,18,19,20,21,22]. Furthermore, the pooled estimate (95% CI) of filter clotting risk showed that RCA is a protective factor for clotting risk (RR = 0.204) with a high study heterogeneity (I^2^ = 63.27%) (Table 4 and Figure 2).

### 3.2. Complications

Six studies describe the complications associated with the CRRT [15,18,19,20,21,22]. Complications described within these studies are detailed in Table 5.

Three studies [15,21,22] (cumulatively counting 315 procedures) describe a significantly higher rate of electrolyte disturbances for patients treated with RCA. In particular, hypokalemia (*p* < 0.05), hypernatremia (*p* < 0.05), hypochloremia (*p* < 0.01), hypomagnesemia (*p* = 0.045), and mild metabolic alkalosis (*p* = 0.036) have been reported.

Three studies [15,19,20] (cumulatively counting for 549 procedures) show a higher transfusion requirement in patients treated with systemic anticoagulation with heparin; nevertheless, only one [20] shows a significant difference (*p* = 0.003) between the two groups. Beyond the results reporting the number of units of red blood cells transfused to the patients, preliminary data are also available for bleeding events and platelet abnormalities. A single study [18] performed on 150 procedures highlights a percentage of severe bleeding events of 32 vs. 30% between patients treated with systemic anticoagulation with heparin and RCA, respectively. Finally, a paper [21] reporting data from 130 procedures describes a significant drop in platelet levels at 72 h from CRRT initiation during systemic anticoagulation with heparin.

### 3.3. Survival

Seven studies (cumulatively counting for 632 patients) report the patients’ survival rate at PICO or hospital discharge, and most of them do not show any differences between both groups. Only one study [12] reporting data from 156 patients shows a statistically higher survival rate in the RCA group (Table 6). Only one study reports long-term patients outcomes about 12 of 28 patients studied: 42% of these did not develop any form of kidney dysfunction; 8% developed low-grade proteinuria; 25% developed CKD; and 25% developed ESKD at least at one-year follow-up, with a mean follow-up length of 3.5 ± 2.0 years [13].

### 3.4. Dialysis Targets

Unfortunately, few studies systematically report dialysis prescriptions. Dialysis targets are detailed in Table 7. Four studies [17,18,21,22] report blood pump flow (Qb) according to mL/min/kg. Qb ranges from 2 to 5 mL/kg/min. Another four studies [13,14,15,20] report blood pump flows in mL/min, and values range from 60 to 96 mL/min. No difference seems to be between RCA and heparin treatments. Finally, all the four studies [13,20,21,22] that describe the heparin dose report a lower dose of 20 UI/kg/h.

## 4. Discussion

Results described in this systematic review show the potential role of RCA in prolonging CL and its superiority with respect to systemic anticoagulation with heparin in decreasing the risk of circuit clotting during CRRT performed in critically ill pediatric patients.

These results are in line with those reported for adult populations. As an example, in a systematic review including 6 randomized controlled trials published in 2012 and cumulatively considering 658 CRRT sessions, Zhang et al. reported a significantly longer circuit life span for treatments performed with RCA [23]. Unfortunately, few studies are currently available aimed at exploring the role of pharmacological strategies in reducing the risk of clotting, specifically in CRRT performed in pediatric patients. It is well known that clotting is a major drawback of extracorporeal treatments. This is true particularly for pediatric patients due to the younger age, the smaller vascular access available for CRRT, and the more limited blood flow achievable for extracorporeal treatment. Furthermore, considering that monitors specifically designed for pediatric treatment and characterized by miniaturized peristaltic pumps are not commonly available, periodic oscillations in the inflow line pressure may lead to excessively negative pressure and frequent treatment interruptions, ultimately causing circuit clotting [24]. For this reason, pharmacologic strategies for anticoagulation seem to be crucial in pediatric patients treated with CRRT to prevent this complication.

Raina et al. have evaluated the safety and efficacy of the extracorporeal anticoagulants in the pediatric CRRT in a systematic review, including any pediatric study reporting data on anticoagulation (heparin, citrate, or prostacyclin) [25]. In this systematic review, including 24 studies, the authors have demonstrated the association between RCA and an average prolonged circuit life with a relatively higher risk of electrolytes imbalance. Even if similar results were obtained in our systematic review, several differences must be remarked. In particular, our analysis was consistently confined to those studies reporting an explicit comparison between RCA and systemic anticoagulation with heparin for the selected outcomes. Hence, we included three more papers [12,13,14] evaluating direct comparison between RCA and heparin, for a total of three hundred and forty patients. Finally, we excluded a paper [25] included in the analysis by Raina et al., because it considered patients up to twenty years old, who have to be considered as adults, especially from the anticoagulation standpoint.

Interestingly, despite the substantial differences in the methodology used, our study reports consistent results with those reported by Raina et al. [25] for bleeding events. In particular, no difference was reported between RCA and systemic anticoagulation with heparin in terms of severe bleeding events. Nonetheless, heparin was associated with a significant risk of a drop in platelet levels and an increased need for transfusions of red blood cell units, probably due to more frequent circuit changes. Indeed, CRRT prescription for younger and smaller children is affected by problems concerning the extracorporeal blood volume, the need for circuit blood priming, and the adaptation of machines designed for adult-sized patients [26]. Blood priming could be necessary when extracorporeal circuit volume exceeds 10–15% of the patient’s blood volume [27]. The more frequent the circuit clotting is and the circuit substitution, the higher the need for blood transfusions and blood units for circuit priming. Furthermore, papers studied reported lower values of heparin dosage than previously published studies, which reported a dosage of 20 UI/kg/h [28,29].

RCA, reducing circuit clotting and prolonging filter life, might thus have a role in attenuating the needs of hemoderivates. Citrate administration can cause metabolic alkalosis and calcium perturbations [28]. Since 2003, Bunchman et al. worked on a simplified protocol to administer citrate anticoagulation, avoiding potential error risks and complications for the patients [30]. Unfortunately, according to our findings, RCA seems to be more commonly associated with electrolyte imbalance and metabolic alkalosis. These results are in line with those reported by Raina et al. [25], where metabolic alkalosis and electrolyte imbalance were reported in 71.4% and 40% of patients treated with RCA (vs. 16.7% and 0% of those treated with systemic anticoagulation with heparin). Similar results are not confirmed for adult patients, where RCA is usually considered safe and effective in maintaining electrolytes and acid-base homeostasis. Indeed, in a meta-analysis of 6 RCTs for a total of 488 adult patients Wu et al. [31], show no statistically significant difference in terms of metabolic alkalosis and electrolyte imbalance between patients treated with different anticoagulation strategies.

Cumulatively considering the effects on circuit outcomes and treatment complications, an economic advantage might be expected in using RCA for CRRT in pediatric patients. Studies on cost analysis including adult patients are available in this field; unfortunately, the same studies are currently lacking for pediatric settings.

Several drawbacks may be recognized in this systematic review. First, most of the considered studies were retrospective in nature, and only one crossover trial was available. Second, the wide heterogeneity in reporting results across different studies (mean and standard deviation or median and interquartile range) did not allow a meta-analysis for outcomes as the circuit life. Third, limited results are reported for patients’ long-term outcomes as renal functional recovery and dialysis dependence between the two groups of anticoagulation strategies.

## 5. Conclusions

Regional citrate anticoagulation could prolong circuit life and decrease the risk of clotting in CRRT performed in critically ill pediatric patients. Although no difference is observed in severe bleeding events, systemic anticoagulation with heparin is associated with a greater reduction in platelet levels during the treatment and with an increased need for transfusions of red blood cells, with respect to RCA. More frequent circuit substitution and the use of blood for circuit priming may explain this phenomenon.

## Figures and Tables

**Table 1:** PICO. PICU: pediatric intensive care unit.

Population	Intervention	Comparison	Outcome	Study Types
Patients < 18 years old admitted in PICU undergoing continuous renal replacement therapy	Use of anticoagulation during CRRT (systemic with heparin or regional citrate anticoagulation)	Heparin vs. RCA	Circuit life (CL) OR clotting rate; Complications (bleeding, blood transfusion rate, electrolyte OR metabolic disturbances); Survival	Prospective and retrospective observational studies; randomized clinical trials

**Table 2:** Characteristics of studies included. Abbreviations: RCA, regional citrate anticoagulation; CL, circuit life.

Source	Study Design	Country	Mean Age (Months)	Sample Size (N) (Citrate)	Sample Size (N) (Heparin)	Outcomes
Chen et al., 2021 [12]	Retrospective Observational	China	48	107	49	Reduced mortality rate with RCA at logistic regression analysis
Buccione et al., 2021 [13]	Retrospective Observational	Italy	48	23	23	RCA as a protective factor for clotting at multivariate Cox regression analysis
Cortina et al., 2020 [14]	Retrospective Observational	Australia	61.2	61	161	No statistical difference in CL between heparin and RCA at multivariate logistic regression analysis
Sik et al., 2019 [15]	Retrospective Observational	Turkey	72	19	26	Median CL was significantly longer for RCA at univariate regression analysis.
Kakajiwala et al., 2017 [16]	Retrospective Observational	United States of America	141.6	26	26	Lower risk of clotting with Heparin anticoagulation at univariate Cox regression analysis.
Miklaszewska et al., 2017 [17]	Retrospective Observational	Poland	116.7	8	32	No differences in the survival rate between the groups
Rico et al., 2017 [18]	Retrospective Observational	Colombia	1 to 216	17	15	Median CL prolonged with RCA at univariate and bivariate regression analysis.
Raymakers-Janssen et al., 2017 [19]	Prospective Observational	Netherlands	15	14	6	Median CL was higher with RCA at log-rank
Zaoral et al., 2016 [20]	Crossover Trial	Czech Republic	84	63	63	RCA prolongs CL at the Wilcoxon paired test
Fernandez et al., 2014 [21]	Prospective Observational	Spain	34.5	12	24	Prolonged CL with RCA at Kaplan–Meier survival analysis
Soltysiak et al., 2014 [22]	Retrospective Observational	Poland	19.7	16	14	Higher CL was observed with RCA at Kaplan–Meier survival analysis.

**Table 3:** Circuit lifetime and clotting rate reported by studies included. RCA: regional citrate anticoagulation.

Source	N Sessions		Circuit Life (h)		Clotting Rate (%)	
	RCA	Heparin	RCA	Heparin	RCA	Heparin
Buccione et al., 2021 [13]	11	72	N/A	N/A	18.2	60.6
Cortina et al., 2020 [14]	132	355	29.3 [25.8–33.1]	23.8 [19.5–29.2]	N/A	N/A
Sik et al., 2019 [15]	44	57	53 [40–70]	40.25 [22.75–53.5]	11.36	26.31
Kakajiwala et al., 2017 [16]	22	51	N/A	N/A	39.2	51
Miklaszewska et al., 2017 [17] (HF20/ST60/ST100)	36	15	41 ± 25.9	33.3 ± 23.8	43.9	29.8
	15	46	57 ± 23.5	53.1 ± 23.8		
	15	23	69.7 ± 8.2	57.2 ± 23.3		
Rico et al., 2017 [18]	80	70	72 [48–96]	18 [12–24]	70	90
Raymakers-Janssen et al., 2017 [19]	105	121	45.2 [37.5–52.8]	21 [14.5–27.5]	17.1	42
Zaoral et al., 2016 [20]	111	111	41 [35–51.75]	36 [31–40]	N/A	N/A
Fernandez et al., 2014 [21]	34	96	48 [31.0–93.7]	31.0 [15.5–71.0]	18.8	76.4
Soltysiak et al., 2014 [22]	43	41	58.04 ± 51.18	37.64 ± 32.51	11.63	34.15

**Table 4:** Pooled estimate of filter clotting risk for regional citrate anticoagulation vs. heparin anticoagulation.

Source	Clotting Rate Difference	95% CI
Buccione	−0.429	0.684–0.175
Sik	−0.150	0.297–0.002
Kakajiwala	−0.101	0.348–0.146
Miklaszewska	0.142	−0.013–0.296
Rico	−0.200	0.323–0.077
Raymakers-Janssen	−0.250	0.364–0.136
Fernandez	−0.584	0.738–0.430
Soltysiak	−0.225	0.399–0.051
**POOLED**	−0.204	0.265–0.144

**Table 5:** Complications according to anticoagulant methods. RCA: regional citrate anticoagulation; RBC, red blood cells.

	Complications		*p*-Value	Complication
Source	RCA	Heparin		
Sik et al., 2019 [15]	7.01%	6.41%	0.956	Metabolic alkalosis
	12.28%	2.56%	<0.05	Hypocalcemia
	14.03%	10.25%	<0.05	Hypernatremia
	0.8 [0.3–2.0]	1.65 [0.5–2.38]	0.32	Units of RBC transfused
Rico et al., 2017 [18]	30%	32.6%	0.605	Severe bleeding events
Raymakers-Janssen et al., 2017 [19]	3 [2.0–5.0]	6.5 [1.5–23.8]	0.12	Units of RBC transfused
Zaoral et al., 2016 [20]	0.17 [0.0–1.0]	0.36 [0.0–2.0]	0.003	Units of RBC transfused
Fernandez et al., 2014 [21]	45.5%	0%	<0.01	Hypochloremia
	27.3%	0%	0.045	Hypomagnesemia
	0%	27.8	0.06	Hypophosphatemia
Soltysiak et al., 2014 [22]	18.75%	0%	N/A	Hyponatremia
Soltysiak et al., 2014 [22]	18.75%	14.3%	N/A	Hypernatremia
	12.5%	21.4%	N/A	Hyperkalemia
	62.5%	28.6%	N/A	Hypokalemia
	43.75%	64.3%	N/A	Hypercalcemia
	43.75%	0%	N/A	Hypocalcemia
	43.75%	42.9%	N/A	Metabolic acidosis
	25%	14.3%	N/A	Metabolic alkalosis

**Table 6:** Survival rate according to anticoagulant methods. RCA: regional citrate anticoagulation; *: statistically significant.

Source	Time-Point	Survival Rate (%)		*p*-Value
		RCA	Heparin	
Chen et al., 2021 [12]	PICU discharge	53.2	34.7	0.031 *
Sik et al., 2019 [15]	PICU discharge	68.42	69.23	0.954
Miklaszewska et al., 2017 [17]	PICU discharge	62.5	34.4	N/A
Rico et al., 2017 [18]	PICU discharge	83.3	81.2	0.859
Raymakers-Janssen et al., 2017 [19]	PICU discharge	50	50	N/A
Fernandez et al., 2014 [21]	PICU discharge	25	25	N/A
Soltysiak et al., 2014 [22]	Hospital discharge	37.5	14.3	N/A

**Table 7:** Dialysis targets. RCA: regional citrate anticoagulation; Qb: blood pump flow.

	Qb (mL/min)		Dialysate (mL/h)		Heparin Dose (IU/kg/h)		Net Ultrafiltration (mL/h)		Replacement (mL/min)		Citrate (mmol/L)	
Source	RCA	Heparin	RCA	Heparin	RCA	Heparin	RCA	Heparin	RCA	Heparin	RCA	Heparin
Buccione et al., 2021 [13]	60 (40–80)	60 (40–80)	400 (200–600)	400 (200–600)	N/A	13.9	40 (25–70)	40 (25–70)	200 (50–400)	200 (50–400)	N/A	N/A
Cortina et al., 2020 [14]	96 (16–400)	96 (16–400)	N/A	N/A	N/A	N/A	N/A	N/A	N/A	N/A	N/A	N/A
Sik et al., 2019 [15]	60 (50–80)	60 (50–80)	700 (500–900)	500 (350–800)	N/A	N/A	N/A	N/A	N/A	N/A	4 (4–5)	N/A
Miklaszewska et al., 2017 [17](HF20/ST60/ST100)	3.5/kg (.5)	3.5/kg (.5)	N/A	N/A	N/A	N/A	N/A	N/A	N/A	N/A	N/A	N/A
	2.1/kg (1.5)	2.1/kg (1.5)	N/A	N/A			N/A	N/A	N/A	N/A	N/A	N/A
	2/kg (.9)	2/kg (.9)	N/A	N/A			N/A	N/A	N/A	N/A	N/A	N/A
Rico et al., 2017 [18]	3.4/kg	3.5/kg	N/A	N/A	N/A	N/A	N/A	N/A	N/A	N/A	N/A	N/A
Zaoral et al., 2016 [20]	90 (70–100)	90 (70–100)	60.34/kg (48.5–118.5)	53.57/kg (38–85)	N/A	15 (13.2–17.9)	N/A	N/A	N/A	N/A	N/A	N/A
Fernandez et al., 2014 [21]	3.2/kg (2–3.8)	5/kg (3.8–5.6)	325 (50–600)	300 (140–500)	N/A	15 (12–25)	75 (50–97.5)	60 (50–90)	50 (0–50)	300 (140–500)	2.6 (2.3–2.9)	N/A
Soltysiak et al., 2014 [22]	3.49/kg ± 1.56	2.88/kg ± 0.80	52.32/kg ± 35.63	71.71/kg ± 39.39	N/A	17 ± 10	N/A	N/A	N/A	N/A	4.05 ± 2.30	N/A

## Supplementary Materials

The following supporting information can be downloaded at: https://www.mdpi.com/article/10.3390/jcm11113121/s1, Table S1: Search strategy for PubMed database; Table S2: Search strategy for CINAHL database; Table S3: Search strategy for EMBASE database; Table S4: Search strategy for Cochrane database; Table S5: Quality evaluation of observational studies according to STROBE reporting guidelines; Table S6: Quality evaluation of Crossover Trial study according to CONSORT reporting guidelines.
