# Supplementary material for: Regional Citrate Anticoagulation and Systemic Anticoagulation during Pediatric Continuous Renal Replacement Therapy: A Systematic Literature Review
Source: J Clin Med. 2022 May 31;11(11):3121. doi: 10.3390/jcm11113121 (PMC9181744; doi:10.3390/jcm11113121)
Supplement: Supplementary file 1 [file jcm-11-03121-s001.zip › jcm-1737764-supplementary.pdf]

Supplemental Material

Table S1 - Search strategy for PubMed Database

**((citrate OR heparin) AND (dialysis OR hemodialysis OR hemofiltration OR kidney replacement therapy OR hemodiafiltration OR continuous renal replacement therapy OR crrt) AND anticoagulation AND (pediatric OR children)) OR ((citrate OR heparin) AND (dialysis OR continuous renal replacement therapy OR crrt OR hemodialysis OR hemofiltration OR kidney replacement therapy OR hemodiafiltration) AND anticoagulation AND (allchild[Filter]).**

Table S2 – Search strategy for CINAHL Database

**(citrate OR heparin) AND (dialy* OR hemodialy* OR hemofiltrat* OR "kidney replacement therapy" OR hemodiafiltrat* OR "continuous renal replacement therapy" OR crrt) AND anticoagulat* AND (pedia* OR paedia* OR child*)**

Table S3 – Search strategy for EMBASE Database

**((citrate OR heparin) AND (dialysis OR hemodialysis OR hemofiltration OR 'kidney replacement therapy' OR hemodiafiltration OR 'continuous renal replacement therapy' OR crrt) AND anticoagulation AND (pediatric OR children)) OR ((citrate OR heparin) AND (dialysis OR 'continuous renal replacement therapy' OR crrt OR hemodialysis OR hemofiltration OR 'kidney replacement therapy' OR hemodiafiltration) AND anticoagulation AND ([newborn]/lim OR [infant]/lim OR [child]/lim OR [preschool]/lim OR [school]/lim OR [adolescent]/lim))**

Table S4 – Search strategy for Cochrane Database

**((citrate OR heparin) AND (dialysis OR hemodialysis OR hemofiltration OR "kidney replacement therapy" OR hemodiafiltration OR "continuous renal replacement therapy" OR crrt) AND anticoagulation AND (pediatric OR children))**

Table s5 - Quality evaluation of observational studies according to STROBE reporting guidelines. (✓: described; ✗: not described)

		Source									
**Item No**		Chen et al. [11]	Buccione et al. [13]	Cortina et al. [14]	Sik et al. [15]	Kakajiwala et al. [16]	Miklaszewska et al. [17]	Rico et al. [18]	Raymakers-Janssen et al. [19]	Fernandez et al. [21]	Soltysiak et al. [22]
Title and abstract	1	✓	✓	✓	✓	✓	✓	✓	✗	✓	✓
**Introduction**											
Background/ rationale	2	✓	✓	✓	✓	✓	✓	✓	✓	✓	✓
Objectives	3	✓	✓	✓	✓	✓	✓	✓	✓	✓	✓
**Methods**											
Study design	4	✓	✓	✓	✓	✓	✓	✓	✓	✓	✓
Setting	5	✓	✓	✓	✓	✓	✓	✓	✓	✗	✓
Participants	6	✓	✓	✓	✓	✓	✓	✓	✓	✓	✓
Variables	7	✓	✓	✓	✓	✓	✓	✓	✓	✓	✓
Data source/ measurement	8	✓	✓	✓	✓	✓	✓	✓	✓	✓	✓
Bias	9	✗	✗	✗	✗	✗	✗	✗	✗	✗	✗
Study size	10	✓	✓	✓	✓	✓	✓	✓	✓	✓	✓
Quantitative variables	11	✓	✓	✓	✓	✓	✓	✓	✗	✓	✓
Statistical methods	12	✓	✓	✓	✓	✓	✓	✓	✓	✓	✓
**Results**											
Participants	13	✓	✓	✓	✓	✓	✓	✓	✓	✓	✓
Descriptive data	14	✓	✓	✓	✓	✓	✓	✓	✓	✓	✓
Outcome data	15	✓	✓	✓	✓	✓	✓	✓	✓	✓	✓
Main results	16	✓	✓	✓	✓	✓	✓	✓	✓	✓	✓
Other analyses	17	✓	✓	✓	✓	✓	✓	✓	✓	✓	✓
**Discussion**											
Key Results	18	✓	✓	✓	✓	✓	✓	✓	✓	✓	✓
Limitations	19	✓	✓	✓	✓	✓	✓	✓	✓	✓	✗
Interpretation	20	✓	✓	✓	✓	✓	✓	✓	✓	✓	✓
Generalisability	21	✓	✓	✓	✓	✓	✓	✓	✓	✓	✓
**Other information**											
Funding	22	✓	✓	✗	✓	✓	✗	✗	✗	✗	✗

Table s6 - Quality evaluation of Crossover Trial study according to CONSORT reporting guidelines (✓: described; ✗: not described)

Source		
**Item No**		Zaoral et al. [20]
Title and abstract	1a	✗
	1b	✓
**Introduction**		
Background and objectives	2a	✓
	2b	✓
**Methods**		
Trial design	3a	✓
	3b	✓
Participants	4a	✓
	4b	✓
Interventions	5	✓
Outcomes	6a	✓
	6b	✗
Sample size	7	✓
**Randomisation**		
Sequence generation	8	✗
Allocation concealment mechanism	9	✗
Implementation	10	✗
Blinding	11	✓
Statistical method	12	✓
**Results**		
Participants flow	13	✓
Recruitment	14	✓
Baseline data	15	✓
Numbers analysed	16	✗
Outcomes and estimation	17	✓
Ancillary analyses	18	✓
Harms	19	✗
**Discussion**		
Limitations	20	✓
Generalisability	21	✓
Interpretation	22	✓
**Other information**		
Registration	23	✗
Protocol	24	✗
Funding	25	✗
